# The Surveillance of Physical Activity, Sedentary Behavior, and Sleep: Protocol for the Development and Feasibility Evaluation of a Novel Measurement System

**DOI:** 10.2196/35697

**Published:** 2022-06-06

**Authors:** Patrick Crowley, Erika Ikeda, Sheikh Mohammed Shariful Islam, Rasmus Kildedal, Sandra Schade Jacobsen, Jon Roslyng Larsen, Peter J Johansson, Pasan Hettiarachchi, Mette Aadahl, Paul Jarle Mork, Leon Straker, Emmanuel Stamatakis, Andreas Holtermann, Nidhi Gupta

**Affiliations:** 1 The National Research Centre for the Working Environment Copenhagen Denmark; 2 Medical Research Council Epidemiology Unit University of Cambridge Cambridge United Kingdom; 3 Institute for Physical Activity and Nutrition Deakin University Melbourne Australia; 4 Occupational and Environmental Medicine Department of Medical Sciences Uppsala University Uppsala Sweden; 5 Institute of Clinical Medicine Faculty of Health and Medical Sciences University of Copenhagen Copenhagen Denmark; 6 Center for Clinical Research and Prevention Bispebjerg and Fredriksberg Hospital Copenhagen Denmark; 7 Department of Public Health and Nursing Faculty of Medicine Norwegian University of Science and Technology Trondheim Norway; 8 School of Allied Health and enAble Institute Curtin University Perth Australia; 9 Charles Perkins Centre Faculty of Medicine and Health University of Sydney Sydney Australia

**Keywords:** accelerometer, thigh-worn, sensor-based, system acceptability, surveillance, physical activity, physical health, physical, sedentary, sedentary behavior, sleep, surPASS, public health

## Abstract

**Background:**

There is increasing recognition of the need for more comprehensive surveillance data, including information on physical activity of all intensities, sedentary behavior, and sleep. However, meeting this need poses significant challenges for current surveillance systems, which are mainly reliant on self-report.

**Objective:**

The primary objective of this project is to develop and evaluate the feasibility of a sensor-based system for use in the surveillance of physical activity, sedentary behavior, and sleep (SurPASS) at a national level in Denmark.

**Methods:**

The SurPASS project involves an international, multidisciplinary team of researchers collaborating with an industrial partner. The SurPASS system consists of (1) a thigh-worn accelerometer with Bluetooth connectivity, (2) a smartphone app, (3) an integrated back end, facilitating the automated upload, analysis, storage, and provision of individualized feedback in a manner compliant with European Union regulations on data privacy, and (4) an administrator web interface (web application) to monitor progress. The system development and evaluation will be performed in 3 phases. These phases will include gathering user input and specifications (phase 1), the iterative development, evaluation, and refinement of the system (phase 2), and the feasibility evaluation (phase 3).

**Results:**

The project started in September 2020 and completed phase 2 in February 2022. Phase 3 began in March 2022 and results will be made available in 2023.

**Conclusions:**

If feasible, the SurPASS system could be a catalyst toward large-scale, sensor-based surveillance of physical activity, sedentary behavior, and sleep. It could also be adapted for cohort and interventional research, thus contributing to the generation of evidence for both interventions and public health policies and recommendations.

**International Registered Report Identifier (IRRID):**

DERR1-10.2196/35697

## Introduction

The surveillance of physical activity, at a population level, is being met with new demands [1]. Surveillance data should now ideally capture physical activities of all intensities across 24 hours, sedentary behavior, and sleep [1]. These data are very difficult to collect accurately using the traditional self-reported measures widely implemented in physical activity surveillance, primarily because habitual activities and sleep are difficult to recall, and therefore, the estimates reported are often incorrect [2].

In an attempt to overcome the limitations of self-reported measures, sensor-based measurements have been implemented in a few select cases of physical activity surveillance [3-7] and in an increasing number of large cohort studies [8-10]. Although the goals of surveillance and cohort research are inherently different—cohort research informs physical activity guidelines, whereas surveillance monitors adherence to guidelines—the challenges they face in implementing sensor-based measurements are similar. These measurements were only possible at considerable expense, with long delays in producing useful data, and with a considerable burden to all users, both those conducting the data collection and those participating in the studies. Burden can be defined as the direct and indirect financial and resource costs of interacting with the system, including the cost of equipment and expert staff, the time required to recruit participants and coordinate a meeting in person, and the time taken to analyze data and produce useful results. Under this definition, the unacceptable burden of traditional sensor-based methodologies can be summarized quite concisely. The sensors are too expensive, recruitment and data collection require a center and involve considerable logistics for participants and staff, and data analysis requires expert personnel. This burden typically results in a lot of time passing before summary reports and findings based on the collected data reach policy makers. Therefore, we need to develop a methodology for implementing sensor-based measurements of physical activity, sedentary behavior, and sleep, which is lower in burden for all users, in line with the World Health Organization’s global strategy on digital health [11].

To date, we have been largely limited by the available technology. However, recent technological advances could enable the development of new systems for the measurement of physical activity, sedentary behavior, and sleep. Technological advances include a new generation of easily attachable, relatively cheap, discrete sensors capable of secure data transfer via Bluetooth connectivity; new smartphone apps for the integration of sensor and participant input, cloud storage, and automated analysis capacity; and web-based applications for the real-time tracking of data collection progression. These technologies potentially give us the ability to rapidly and automatically analyze data and provide useful, timely feedback to all users in a manner compliant with current data privacy regulations. Additionally, such technologies will increase the potential for collecting and harmonizing data within and across countries in the future, if adapted as a standardized methodology for accelerometry data collection. Thus, exploiting these new technological developments will be the first step toward improving the way we collect accelerometry data at scale.

The surveillance of physical activity, sedentary behavior, and sleep project team (SurPASS) envisions incorporating this new generation of technical solutions and infrastructure in the development of a system for sensor-based surveillance that can be used globally. We see the current protocol as the first step, where we document a method for the development and evaluation of a sensor-based system for surveillance among working age adults at a national level, in Denmark. In this protocol, we will address three aims:

1. Defining the user specifications of such a system.
2. Developing a system through a process of iterative evaluation and redesign.
3. Evaluating the feasibility of this system in a national surveillance program.

## Methods

### Overview

In this section, we describe the components for the SurPASS system, the establishment of user groups, and a plan for system development and a feasibility evaluation. The project team consists of an international group of multidisciplinary researchers and an industrial partner based in Copenhagen, Denmark (SENS Innovation ApS). SENS Innovation ApS will provide sensors, patches, and technical expertise. The target population of this first step toward achieving the SurPASS project team’s vision is adults of working age in Denmark. In describing our method, we define users as any person who uses the system for some purpose. Exclusion criteria for users include already receiving a pension, being on maternity/paternity or sick leave, and anyone who suffers from an allergy to adhesive plasters.

### Ethics Approval

The scientific ethics committee for the Capital Region of Denmark (journal number: 20030293) approved the SurPASS project, which will be conducted in accordance with the Declaration of Helsinki. All participants will be asked to provide informed consent before participation.

### SurPASS System Components

#### A Wearable Sensor

An easily attachable Conformité Européenne–approved triaxial accelerometer (SENSmotionPlus, SENS Innovation ApS) will be used in the development of the SurPASS system. The SENSmotionPlus is a discrete, lightweight accelerometer (47 mm length × 22 mm breadth × 4.5 mm thickness; 7 grams), which is waterproof and has a memory capacity of approximately 4 days when sampling at 25 Hz. However, integrated Bluetooth data-transfer technology (2.4 GHz low-energy transfer) enables data transfer when in Bluetooth range of a user’s smartphone, thus ensuring that the memory capacity will not be exceeded. The sensor has primarily been used in clinical settings to date [12] and has yet to be tested in free-living settings. A validation plan for the use of SENSmotionPlus in free-living settings is presented later.

#### A Smartphone App

A smartphone app will be developed to provide instructions to users regarding sensor attachment and calibration, while allowing the user to register work and sleep time. Data from the sensor will be transferred through the app to a cloud-based back end. Individualized feedback on daily activity types and durations will then be made available for participants through the smartphone app. The app is compatible with both Android and iOS operating systems.

#### A Back-end Infrastructure

A secure, back-end infrastructure developed by SENS Innovation will be adapted to meet the requirements of SurPASS. This back-end infrastructure is compliant with the General Data Protection Regulation (GDPR). It will also facilitate functions such as automated data cleaning, storage, and the integration of processing software.

#### A Processing Software

A new software flow will be created based around the validated MATLAB-Acti4 software [13]. Acti4 has been developed and validated for use with thigh-worn accelerometer data [13,14]. However, the current Acti4 flow requires considerable time for file conversion, manual data cleaning, and processing. This software flow needs to be easier to use, more automated, and capable of meeting the requirements of the back-end infrastructure developed by SENS Innovation. Most critically, the software must maintain the validated Acti4 definitions of physical activity types and postures (eg, sitting, standing, lying down, walking, running, cycling), which are defined for any epoch length >1 second [13]. In addition, it should be capable of differentiating lying down from sitting using a single accelerometer [15].

#### A Web-Based Application for Administrators

A web-based application will be developed to cover all core administrative tasks such as participant registration, monitoring the status of sensors, tracking when notifications need to be sent to participants, and facilitating downloading raw and processed data that can be used for alternative analysis methods and further research.

### Conceptual Framework

**Figure 1:**
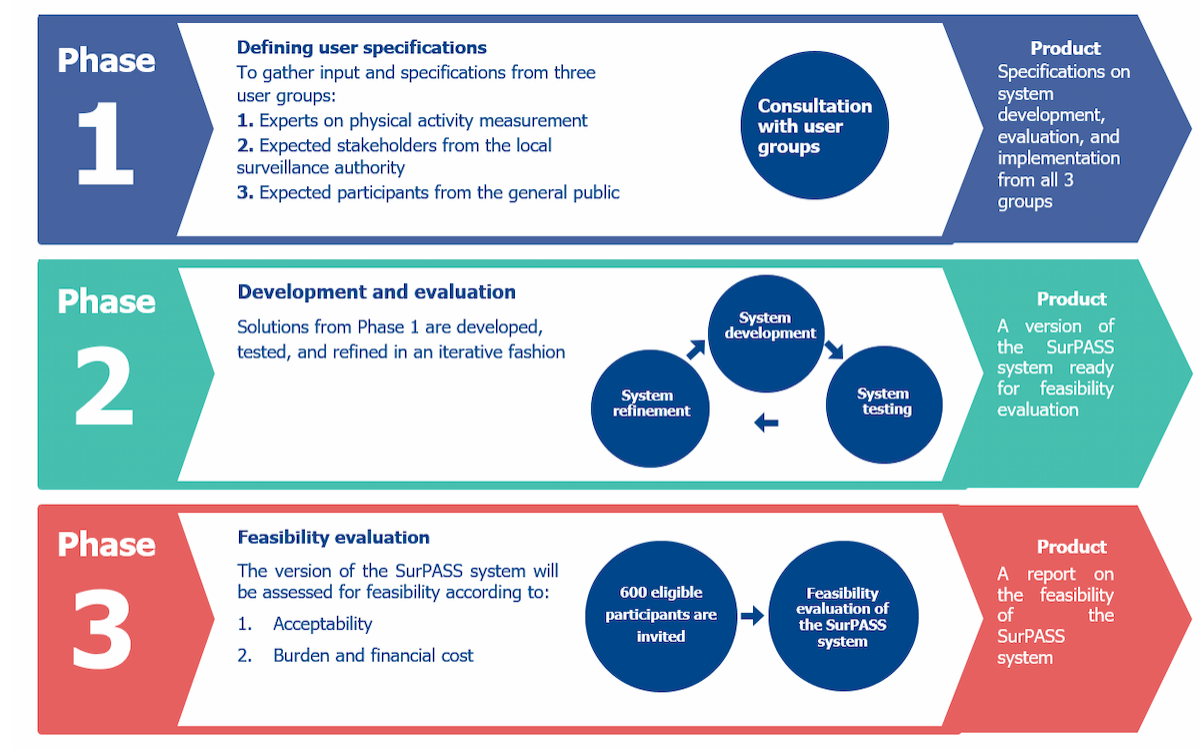
The 3-phase development and evaluation plan for the SurPASS system. Phase 1 is aimed at defining user specifications and outlining a system implementation plan; phase 2 is aimed at developing, evaluating, and refining the SurPASS system based on this plan; and phase 3 is aimed at evaluating the feasibility of the refined system. SurPASS: surveillance of physical activity, sedentary behavior, and sleep.

We will develop the system and evaluate its implementation according to the user-centered design framework [16] (UCD) and a 3-phase plan outlined in Figure 1. UCD refers to an iterative design process, where design decisions are based largely on users’ needs and specifications [16]. Under this framework, the “user” is defined as any person that “uses” the system for some purpose [16]. We plan to establish 3 groups of prospective users to aid us throughout the development and evaluation process. The first group (group 1) will consist of leading experts in the field of physical activity measurement, who will ensure the high scientific quality of the project evaluation, share practical experience regarding sensor-based data collection, and outline their needs as potential users of the system. These experts will be chosen within Denmark and internationally, based on their experience with sensor-based measurements of physical activity and sedentary behavior. The second group (group 2) will consist of local surveillance authority representatives and union representatives (ie, employer and employee unions), who are considered as critical stakeholders for implementation in the Danish social context, largely because the SurPASS system will necessitate measurement during working hours and thus present ethical concerns. The inclusion of these stakeholders is vital for ensuring the social acceptability of the SurPASS system in Denmark. The third group (group 3) will consist of members of the general public. Each group will provide their input on SurPASS system components (eg, smartphone app and the personalized feedback). All groups will be established using existing networks at the National Research Centre for the Working Environment, Copenhagen, Denmark.

### Study Phases

#### Phase 1: Defining User Specifications

**Figure 2:**
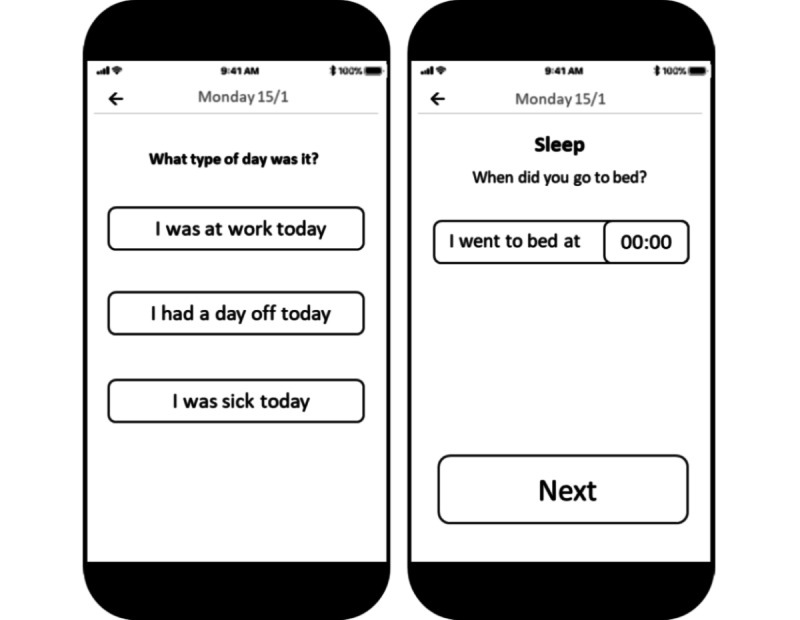
An example of a wireframe outline of a system component. Users will be asked to "think aloud" regarding the appearance and functionality of each component.

User input from the 3 user groups will inform the system specifications. As a starting point, the SurPASS team will develop wireframes (ie, initial outlines) of the SurPASS system’s components to present to user groups for input and feedback (Figure 2). Input and specifications from groups 1 and 2 will be gathered through stakeholder meetings. The initial meeting will be centered around the question, “What would be the requirements for developing and implementing a valid, low-cost, large-scale measurement system with low demands on all users?” Five subsequent biannual meetings will be aimed at seeking expert guidance and input, while also serving as a forum for updates on the project’s progress. Input and specifications from group 3 will be gathered through online video consultations following a “think-aloud” format. Two SurPASS team members will take notes while the initial nonfunctional wireframe outlines of system components are presented on the screen to members of group 3. They will be asked to think aloud, giving their opinion on the appearance and functionality of each component (eg, smartphone app, sensor attachment instructions) as they interact with it. The notes taken during these sessions will be coded and translated into concrete solutions for system improvements by the SurPASS team. Coding will be done according to the severity of the issue concerning the app functionality (ie, green=no issues, blue=a minor problem, yellow=a serious problem, red=a critical problem).

The product of phase 1 will be the collated input and specifications from all user groups. This information will be used to develop the system components and to outline how the system should be implemented. Phase 1 must produce functional system components, which can then be evaluated and refined in phase 2 (eg, an interactive smartphone app).

#### Phase 2: Development and Evaluation

Development will begin, using translated solutions from phase 1 as a starting point. These solutions will be evaluated and then further developed. Members of group 3 (the general public) will be involved in the evaluation in phase 2.

##### Iteration 1: Web Consultation

A functional browser-based interactive wireframe of the smartphone app—designed based on the specifications in phase 1—will be presented to 3-5 members of group 3 during online web consultations in a similar setting to phase 1. Here, emphasis will be placed more on the component functionality as the level of interactivity increases. Again, color-coded (ie, green, blue, yellow, red) notes will be translated into concrete changes in app design, leading to a beta version of a smartphone app available for public download. This beta version will be used in the evaluation during iteration 2.

##### Iteration 2: Evaluating System Acceptability

Using the beta smartphone app, we will evaluate the SurPASS system according to the System Acceptability framework outlined in usability engineering [17]. We will test aspects of acceptability, including system usability using the System Usability Scale [18], the utility of the sensor and the smartphone app, and the practical acceptability of the system processes including the information provided on participation, attachment, personalized feedback, and the experience of participation.

**Figure 3:**
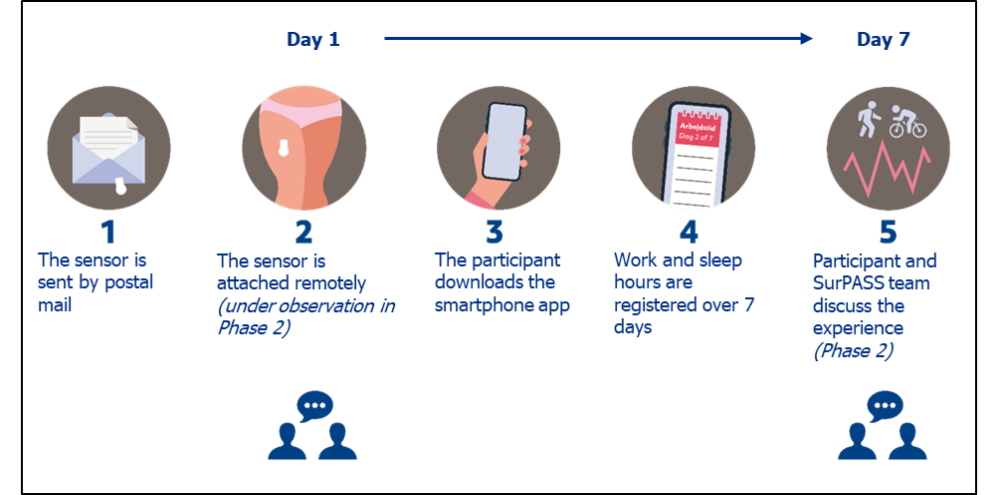
The planned procedure for system acceptability testing in iteration 2 of phase 2. Users from group 3 will receive a package containing a sensor and attachment material via postal mail. During an online video consultation with SurPASS team members, users will attach and initiate the sensors to begin the measurement of physical activity, sedentary behavior, and sleep. On day 7, a second web consultation will be held to discuss the participants' experience.

The evaluation will be conducted according to the procedure summarized in Figure 3. Briefly, a further 10-12 new members of group 3 will be asked to attach and wear a sensor over 7 consecutive days, while logging their work and sleep time in the beta smartphone app. Through online video meetings on days 1 and 7, members of the SurPASS team will observe and rate the use of provided instructions, the success of remote attachment, and the experience and expectations of participation, including users’ responses to personalized feedback. The same process of think-aloud consultations and the color-coding method will be used in the consultation on day 1. In the consultation on day 7, a semistructured interview will be conducted. We chose a 7-day measurement window to capture a full working week and 2 weekend days. There is little consensus on the most representative time window; most studies around the world have chosen a 7-day measurement window for sensor-based measurements. The work of Bergman and Hagströmer [19] suggests that the size of the sample, not the length of the measurement window, is most important for reducing the standard error of the mean. The SurPASS team will use this information to refine the SurPASS system for use in the prefeasibility pilot in iteration 3.

##### Iteration 3: Pilot Evaluation

**Figure 4:**
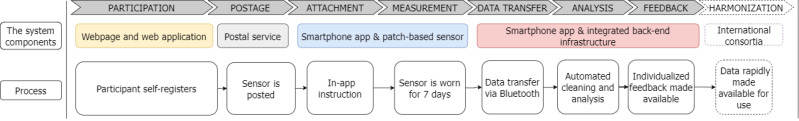
The SurPASS system implementation outline, including the required system components and processes. The participant registers, receives a package, attaches and wears a sensor for 7 days, and registers sleep and work time. Data are then automatically transferred to the cloud for analysis and storage. At the end of the measurement, feedback on the participant’s daily activities is rapidly produced for all users.

A pilot evaluation will be conducted to test all processes of the SurPASS system, developed and refined in iteration 2, to ensure that the system is ready for the feasibility evaluation in phase 3. A procedure following the full system implementation outline (Figure 4) will be conducted among a further 30-50 new members of group 3. A semistructured interview will be conducted after the 7-day test period, to explore the participants’ experience of interacting with the system. The results of the pilot evaluation will be used to solve any unforeseen issues in the system implementation outline (Figure 4) before the feasibility evaluation in phase 3. Any changes in the procedure will be reported accordingly.

By the end of phase 2, we expect to have tested components of the system on between 50-100 members of group 3.

#### Phase 3: Feasibility Evaluation

The feasibility of the SurPASS system will be assessed based on acceptability and user burden. Acceptability is defined as both the *practical*—the success of critical processes in the system (eg, postage of sensors)—and the *social*—the willingness of participants to interact with the system (eg, recruitment) and their level of satisfaction with their experience. Based on the rules of thumb presented in a recent guidance on using pilot studies to inform the design of intervention trials with continuous outcomes, a sample size of approximately 200-250 participants will be required for our evaluation [20]. Since previous large-scale, sensor-based data collection studies in a European context reported recruitment rates of between 31% and 68% [7,9], we estimated that approximately 600 participants should be contacted. Prospective users (ie, members of the general public) will be recruited through the established recruitment cycle of a local surveillance authority in Denmark. A feasibility evaluation will be conducted by applying methods from the updated framework for evaluating complex interventions, including an evaluability analysis of feasibility outcome measures [21] and a traffic light system based on predefined criteria [22]. Under the traffic light system, red indicates an unacceptable performance on a feasibility outcome measure, amber indicates a potentially acceptable outcome if amendments are made, and green indicates an acceptable performance. The criteria for the traffic light system will be defined through an evaluability assessment [22] with groups 2 and 3.

All data will be collected and stored in compliance with GDPR. Participants will be anonymized by assigning identification numbers upon registration to the study. These identification numbers will then be transmitted to a data manager who will assign new variable names, returning a data set to researchers in a pseudorandomized version. While stored on the patch-based accelerometer, data will be encrypted according to the industrial standard AES-128. Transfer to cloud storage will be end-to-end encrypted, using a service provided by Amazon Web Services (AWS GDPR Data Processing Addendum). The stored data will be encrypted according to the industrial standard AES-256. Moreover, the data hosted by AWS and collected within the European Union (EU) will remain within EU territories and will not be accessed outside the EU. Data in the AWS database will only be accessed via a web application with access restricted to the SurPASS team. The web application will require a special login via an industry standard secure “https” connection.

### Sensor Validation

Validation will be achieved by comparing the time spent on various physical activities and sedentary behaviors as measured using SENSmotionPlus against those measured by the Axivity AX3 (Axivity Ltd) and ActivPAL Micro4 (PAL Technologies) accelerometers in controlled and semicontrolled settings on the same participants. Video recording will be used as the gold-standard observation. This data will be analyzed using the SurPASS software flow that will be created around the MATLAB-Acti4 software [13].

## Results

The project started in September 2020. Phase 2 was completed in February 2022 and phase 3 began in March of 2022. Findings will be published in 2023. The proposed system implementation in phase 3 is outlined below (Figure 4).

1. Participants will first register to participate via an online registration system.
2. When registration is completed, the sensor and instructions on using the system will be placed in a package and sent to the participant via postal mail.
3. Once the participant receives the package, they will download the smartphone app and follow the in-app instructions regarding the attachment of the sensor.
4. Once attached and connected to the app via Bluetooth, the sensor will record thigh movements for 7 days, uploading data regularly.
5. Data will be transferred to the back-end infrastructure, and will undergo cleaning, batch processing, and storage.
6. At the end of the measurement period, the participant will enter their last day of diary information before receiving feedback on their physical activity, sedentary behavior, and sleep during the measurement period.

## Discussion

This protocol describes the developmental process that is the first step toward the next generation of surveillance tools for physical activity, sedentary behavior, and sleep. In designing the protocol, we have attempted to meet a number of new demands faced by traditional sensor-based measurement methodologies at a large scale, namely, a secure, sensor-based system that is easy to use for all users, while ensuring the rapid collection of accurate and useful data. In the process of producing the system implementation outline (Figure 4), we identified 3 key areas of risk.

First, we identified the risk of low recruitment and representative participation. Previously, sensor-based measurement on a large scale, whether it be for surveillance or cohort purposes, has struggled to recruit representative samples of sufficient size [23]. Through the early establishment and engagement of user groups, this risk can be mitigated, particularly by designing a system that overcomes barriers to use and fulfills user needs and desires. As this risk is context-dependent (eg, country, culture, institutions), future administrative users (groups 1 and 2) will need to consider how to ensure sufficient participation in the context where the system is implemented, and to adapt their approach accordingly.

Second, we identified the risks of remote sensor attachment, poor sensor utility, and poor adherence. Our system implementation outline relies on (1) the participant being able to attach the sensor easily, (2) the sensor being appropriate for free-living measurement, and (3) a high participant motivation to adhere to the procedure over several days. We plan to seek early input from the participant group (group 3) on the ease of comprehension of attachment instructions, and have repeated data collection throughout phases 1-3 to improve this aspect. Further, we will use phase 2 to provide an indication of the utility of the sensor in free-living settings and to maximize the usability of the system, which can encourage better adherence.

Third, with such a complex technological system, there will always be a risk of technological failure. The system implementation outline relies heavily on technical infrastructure for data transfer (from the sensor through the app to the cloud storage), automated analysis, and the provision of feedback. If any of these steps fail, we would risk losing data. We plan to integrate existing tried-and-tested technical infrastructure to mitigate this risk.

The strength of this protocol is the continuous inclusion of stakeholders throughout the development and evaluation of the system. Further, the development and evaluation of the system will be conducted in iterative cycles, allowing for continual improvement and incorporation of user input. A limitation of the current protocol is that the evaluation of the system will be limited to the social and cultural context that it is tested in. Each of the challenges and risks highlighted above will vary in degree depending on where in the world the system is tested and the culture of that place. Thus, the evaluation of whether the system is feasible or not will only be applicable in a Danish context until tested elsewhere. Future projects could consider including methods such as ecological momentary assessment to better understand the influence of context [24]. We have also opted to implement a single thigh-worn sensor. This placement has some limitations and the use of a single sensor does not allow for the capture of important physical activities such as awkward postures (ie, those deviating considerably from neutral positions)—for example, elevating arms above shoulder level and bending of back, kneeling, and squatting. Finally, in this step, we focus only on adults of working age, limiting our findings to this population.

The SurPASS system developed in this project will be just the first step toward achieving the SurPASS team’s vision. Further evaluation will undoubtedly be required to consider and improve on any transparency, accessibility, scalability, replicability, interoperability, privacy, security, and confidentiality issues that may arise as the system is adapted and challenged in new contexts (eg, low- to middle-income countries). In this first step, we consider the privacy, security, confidentiality, and transparency issues within the Danish context through the development of data usage and licensing agreements, as well as provide indications of future scalability through cost analysis. If the SurPASS system were feasible, it would represent a huge leap forward in the process of developing surveillance tools to meet the new demands on physical activity surveillance. The SurPASS system could facilitate the production of more accurate and useful data, which can be rapidly provided to key stakeholders once the validity of the sensor has been fully established. We believe that by developing a system that is low in burden, the prevalence of sensor-based measurement will increase, perhaps reaching population groups that are seriously underrepresented currently (eg, low- to middle-income countries, lower socioeconomic status settings). A higher prevalence of sensor-based measurements would also lead to increased public awareness regarding the importance of daily physical activity, sedentary behavior, and sleep (eg, through the rapid provision of accurate feedback). However, feedback to users is something that will need to be continually optimized as we gather better user information on how feedback is perceived to ensure it is relevant for users. Finally, many of the challenges encountered in the surveillance of physical activity are also faced by cohort and interventional studies, particularly burden and cost. The SurPASS system could be adapted for cohort and intervention research, further expanding the capacity of research programs and consortia [25,26] to provide high-quality evidence to inform policy and practice. The SurPASS project started in September 2020 and is currently in phase 2. The first results of the development and evaluation will be available in 2022, with the results of the feasibility evaluation made available in 2023.

The protocol presented describes a system that, if feasible, could act as a catalyst toward large-scale, sensor-based surveillance, thus taking the first step toward advancing physical activity, sedentary behavior, and sleep research and elevating evidence-informed policy and practice**.**

## Abbreviations

AWS: Amazon Web Services
EU: European Union
GDPR: General Data Protection Regulation
SurPASS: surveillance of physical activity, sedentary behavior, and sleep
UCD: user-centered design
